# Supplementary material for: Diffusive spreading across dynamic mitochondrial network architectures
Source: Proc Natl Acad Sci U S A. 2026 Apr 9;123(15):e2523913123. doi: 10.1073/pnas.2523913123 (PMC13079940; doi:10.1073/pnas.2523913123)
Supplement: Supplementary file 1 — Appendix 01 (PDF) [file pnas.2523913123.sapp.pdf]

Supporting Information Text

1. Supplemental Methods

A. Exact solution for static physical networks. For a stationary network structure, the total material content at steady-state can be computed analytically by solving the diffusion equation on each linear segment, then linking these solutions together at the junctions. The segment containing a source unit is separated into two segments on either side of the source. The diffusion equation and corresponding boundary conditions for each segment is:

$$\begin{aligned} D_p \frac{d^2 c_n(x)}{dx^2} - k_d c_n(x) = 0, \\ c_{n_1^i}(x_{n_1^i}) = c_{n_2^i}(x_{n_2^i}) = c_{n_3^i}(x_{n_3^i}), \\ \sum_{j=1}^{\deg(i)} \left. \frac{dc_{n_j^i}}{dx} \right|_{x_{n_j^i}} = 0 \end{aligned} \qquad [S1]$$

where n_j^i is the j-th edge connected to node i and $x_{n_j^i}$ is the value of x on that edge at node i. For each segment, $0 < x < \ell_{n_j^i}$ and $x_{n_j^i} \in \left\{0, \ell_{n_j^i}\right\}$, where $\ell_{n_j^i}$ is the segment length. The middle equation defines continuity of the concentration fields, and the final equation conserves flux. This system has general solutions:

$$c_n(x) = A_n \cosh(x/\lambda_p) + B_n \sinh(x/\lambda_p)$$

allowing us to rewrite the boundary conditions as a large matrix equation $M\vec{\alpha} = \vec{v}$, where $\vec{\alpha}$ is a stacked vector of coefficients A_n, B_n, and $\vec{v}$ is zero except for boundaries of the source unit. The total material in the network is found by integrating along all segments m outside of the source unit, to yield $S = \sum_m \int_0^{\ell_m} c_m(x)\, \mathrm{d}x$.

Because the material delivered will depend on the choice of source unit within the network, we repeat this procedure for all possible choices of source unit and average the values of S. This result is then averaged over each replicate snapshot to give the exact solution (solid curves in Fig. 2).

B. Continuum Approximation for Static Physical Networks. We model highly-connected, stationary, physical network structures as an effective d-dimensional sphere through which diffusive material spreads from a localized source.

B.1. Selection of the effective domain size R_n**.** To extract the appropriate size of the continuum domain, we use the graph dimension d and the mean graph distance between paris of nodes $\langle r_g \rangle$ in a given network. As we are approximating the network by a d-dimensional sphere, the mean distance between points in that sphere (1) should be equal to the mean graph distance. This yields the relationship:

$$\begin{aligned} R_n = \frac{(2d+1)\langle r_g \rangle}{2d\beta_d}, \\ \beta_d = \frac{2^{d+1}\Gamma^3(d+1)}{(d+1)\Gamma^2(\frac{d+1}{2})\Gamma(2d+1)} \end{aligned} \qquad [S2]$$

However, for dimension less than 2, where diffusive search is compact and geometry-dependent (2), the position of the source within the domain becomes important.

For the linear case ($d = 1$), specifically, a source point selected at random on a line of length $2R_n$, will on average be a distance $R_n/2$ from the nearest end. The steady-state distribution profile $c(x)$ from this source can be found analytically by solving the steady-state equation $D_p \frac{\partial^2 c}{\partial x^2} - k_d c = 0$ with reflecting boundaries at $[0, 2R_n]$ and a fixed point $c(R_n/2) = 1/\ell_0$. This gives the steady-state material content in the domain:

$$S = \frac{\lambda_p}{\ell_0}\left[\tanh\left(\frac{R_n}{2\lambda_p}\right) + \tanh\left(\frac{3R_n}{2\lambda_p}\right)\right] \qquad [S3]$$

An alternate approach is to solve for $S^{(1)}$: the material delivered from a central source in a linear domain of radius $R^{(1)} = R_n/2$, and separately $S^{(2)}$: material delivered from a central source in a domain of radius $R^{(2)} = 2R_n - R_n/2$. Averaging these results together gives the exact expression in Eq. S3.

Analogously, for the d-dimensional case, a source point selected at random in a hypersphere of radius R_n will be at a distance of $R^{(1)} = 1/(d+1)R_n$ from the boundary. We compute $S^{(1)}$: the total material delivered from a central source in a hypersphere of radius $R^{(1)}$. Separately, we compute the result $S^{(2)}$ for a hypersphere of radius $(R^{(2)})^d = 2(R_n)^d - (R^{(1)})^d$. The average d-dimensional volume of the two hyperspheres is thus equal to the total volume. The two resulting solutions for delivered material are averaged together to give $S = (S^{(1)} + S^{(2)})/2$. Although this is not an exact solution for $d > 1$, it provides a good approximation for material content in simulated networks, where source points are selected uniformly to start anywhere on the network (Fig. 2, dashed-square curves).

B.2. Solving the continuum d-dimensional model (Eq. 1). Starting with the general solution for the concentration (3):

$$c(r) = Ax^{\nu} I_{\nu}(x) + Bx^{\nu} K_{\nu}(x), \tag{S4}$$

where I_{ν}, K_{ν} are the modified Bessel Functions, we first apply the boundary conditions to solve for the constants A, B as follows. The reflecting outer boundary sets:

$$\begin{aligned} \left.\frac{\partial c}{\partial r}\right|_{R_n} = \frac{\sqrt{d}}{\lambda_p} \left.\frac{\partial c}{\partial x}\right|_{x_2} &= Ax_2^{\nu} I_{\nu-1}(x_2) - Bx_2^{\nu} K_{\nu-1}(x_2) = 0 \\ \rightarrow \quad \frac{B}{A} &= \frac{I_{\nu-1}(x_2)}{K_{\nu-1}(x_2)} \end{aligned} \tag{S5}$$

and the fixed-concentration inner boundary sets:

$$\begin{aligned} c(a_n) = c(x_1) = C_0^{(d)} &= A\left(x_1^{\nu} I_{\nu}(x_1) + \frac{B}{A} x_1^{\nu} K_{\nu}(x_1)\right) \\ \rightarrow \quad A &= \frac{C_0^{(d)} x_1^{-\nu}}{I_{\nu}(x_1) + \frac{B}{A} K_{\nu}(x_1)}, \end{aligned} \tag{S6}$$

where $C_0^{(d)}$ is related to the linear concentration of the fixed unit, h_0, as $M_d a_n^d C_0^{(d)} = \ell_0 h_0 = 1$, and M_d is the volume of a d-dimensional unit sphere (i.e. $\frac{4}{3}\pi$ in 3D). The total material delivered is then found through integration of the concentration field over space:

$$\begin{aligned} S_d =& U_d \int_{a_n}^{R_n} r^{d-1} c(r) dr = U_d \left(\frac{\lambda_p}{\sqrt{d}}\right)^d \int_{x_1}^{x_2} x^{d-1} c(x) dx \\ =& U_d \left(\frac{\lambda_p}{\sqrt{d}}\right)^d A \left[-x_1^{1-\nu}\left(I_{\nu-1}(x_1) - \frac{B}{A} K_{\nu-1}(x_1)\right)\right] \end{aligned} \tag{S7}$$

where U_d is surface area of a d-dimensional unit sphere (i.e. 4π in 3D) and the x_2 term vanishes by the outer boundary condition. Substituting in the definitions of $A, C_0^{(d)}$, and B/A we arrive at Eq. 1:

$$\begin{aligned} S_d =& U_d \left(\frac{\lambda_p}{\sqrt{d}}\right)^d x_1^{1-2\nu} \left(\frac{1}{M_d a_n^d}\right) \frac{\left[-I_{\nu-1}(x_1) + \frac{B}{A} K_{\nu-1}(x_1)\right]}{\left[I_{\nu}(x_1) + \frac{B}{A} K_{\nu}(x_1)\right]} \\ =& \frac{d}{x_1} \frac{\left[-I_{\nu-1}(x_1) + \frac{I_{\nu-1}(x_2)}{K_{\nu-1}(x_2)} K_{\nu-1}(x_1)\right]}{\left[I_{\nu}(x_1) + \frac{I_{\nu-1}(x_2)}{K_{\nu-1}(x_2)} K_{\nu}(x_1)\right]} \end{aligned} \tag{S8}$$

since U_d/M_d is simply the dimension d (4).

C. Mean-field model for social networks. For social networks consisting of identical diffusing units that exchange contents upon interaction, we develop a continuum field-based model for the spatial spreading of material. This model can apply to small clusters as well as completely fragmented units, so long as the material distribution within each cluster is approximately homogeneous.

C.1. Model for spatial material concentration (Eq. 2). We derive the dynamic equation of a spatial mean concentration field, $h(\vec{r}, t)$, defined as the average material per unit at position $\vec{r}$ relative to the source cluster at time t. For uniform diffusing clusters in 3D, we assume spherical symmetry and express the concentration field as a function of the radial coordinate r. The material is carried by clusters with relative diffusivity D and decays at rate k_d, giving a simple reaction-diffusion equation for positions outside the contact zone ($r > b$):

$$\frac{\partial h(r,t)}{\partial t} = D\nabla^2 h(r,t) - k_d h(r,t) \tag{S9}$$

Material exchange between non-source clusters has no effect on the average material concentration at the given position.

Two adjustments are made to Eq. S9 when clusters are within the contact volume v_c ($a < r < b$), where they are able to undergo fusion with the source cluster. First, we assume that the contact region is very thin relative to the domain size, so that the concentration field can be regarded as a spatially uniform value: $h_c(t)$. Material leaves the contact zone via diffusive flux through its outer boundary. This flux also forms the boundary condition at $r = b$ for Eq. S9. The spatial density of material is ρh, where ρ is the density of mitochondrial units. The overall current leaking out of the zone can then be expressed as:

$$I = -D\rho \left.\frac{\partial h(r,t)}{\partial r}\right|_b (4\pi b^2) \tag{S10}$$

The per-unit loss of concentration is $I/(\rho v_c)$, as we must divide the total escaping concentration by the number of units in the contact volume.

An additional adjustment accounts for the injection of material into the network upon fusion through the term $+k_u(h_0\ell_0 - h_c)$, where k_u is the rate for each cluster to fuse with the source. This term arises from the fact that whenever a cluster fuses with the source cluster, both clusters leave the interaction with concentration $h_0 = 1/\ell_0$, meaning that the material per unit in the non-source cluster increases by $(h_0\ell_0 - h_c)$. With these modifications, we arrive at the dynamic equation for material per unit within the contact zone:

$$\frac{dh_c(t)}{dt} = k_u(h_0\ell_0 - h_c(t)) - \frac{I}{\rho v_c} - k_d h_c(t) \quad \text{[S11]}$$

At steady-state, the time derivatives in Eq. S9, S11 are set to 0 to solve for the spatial profile $h(r)$, as described below.

C.2. Selection of the steric and contact radii, a, b, effective fusion rate, k_u, and relative diffusivity, D. The continuum model in the social network regime requires defining inner and outer radii a, b for interacting clusters. Since the inner radius a represents a steric exclusion distance, we set $a = 2R_g$, where R_g is the average radius of gyration for clusters in the network. Rather than defining b directly, we set the total volume v_c available for fusion to a cluster by multiplying the average number of nodes available for fusion (those with degree below 3) by the contact volume per node. The contact volume per node describes the volume within which two individual nodes can undergo fusion; it is computed in detail in Ref. (5). This procedure fixes b through the relationship $v_c = \frac{4}{3}\pi(b^3 - a^3)$.

The effective fusion rate is also dependent on the number of nodes available for fusion. In our simulations, we have separate orientation-dependent rates for tip-tip (between two degree-1 nodes) and tip-side (between a degree-1 and degree-2 node) fusion: $k_{u1}(\Theta)$ and $k_{u2}(\Theta)$. Here Θ encompasses the angle(s) between the 2 (tip-tip) or 3 (tip-side) units involved in the fusion event. For further details and an exploration of the effect of orientation sensitivity on network structure, see Ref. (5). The effective rate k_u is calculated as a weighted sum of these two fusion types:

$$k_u = \langle k_{u1}(\Theta)\rangle \cdot x_1\left(\frac{x_1}{x_1 + x_2}\right) + \langle k_{u2}(\Theta)\rangle \cdot \frac{2x_1x_2}{x_1 + x_2} \quad \text{[S12]}$$

where the angle brackets denote averaging over orientations for a pair of nodes, given that they are close enough in space to fuse (within the contact volume per node) and x_1, x_2 denote the average number of degree 1 and 2 nodes per cluster, respectively. Since all nodes in the incoming cluster have a chance to fuse with the source cluster while inside v_c, the tip-tip rate is multiplied by the number of degree-1 nodes in the incoming cluster and the probability to be near a degree-1 node from the source cluster, given a position inside v_c. The tip-side rate is weighted analogously, but contains two contributions. First, we include the number of degree-1 nodes in the incoming cluster multiplied by the probability to be near a degree-2 node from the source cluster. Second, we have the number of degree-2 nodes in the incoming cluster multiplied by the probability to be near a degree-1 node from the source cluster. Further details of the fusion and fission model and its dependence on the orientation and position of interacting units can be found in Ref. (5).

Relative diffusivities are obtained as follows. First, the mean-squared displacement Δx^2 is measured for each unit over lag time $\Delta t = 1.68$. Next, we record the instantaneous diffusivity $(\Delta x^2/(6\Delta t))$ and size of the cluster to which each unit belonged at the beginning of each time interval. For a cluster of a given size m, we define its diffusivity $D^{(m)}$ as the (time- and ensemble-) average value among all units that started in a cluster of that size. Finally we calculate the mean cluster diffusivity $D_n = \sum_m D^{(m)} N_m / \sum N_m$ where N_m is the number of clusters of size m in the simulation. Here, n is the average size of cluster for a randomly selected unit: $n = \sum m^2 N_m / \sum m N_m$. The relative diffusivity D used in the analytic model is $D = 2D_n$ as explained in the main text.

C.3. Solving the mean-field model (Eq. 2,3). We begin by writing down the general solution at steady-state, $h(r) = \frac{1}{r}\left(Ae^{r/\lambda} + Be^{-r/\lambda}\right)$ where $\lambda = \sqrt{D/k_d}$, with D the relative cluster diffusivity. Applying the outer boundary condition gives:

$$\begin{aligned} \left.\frac{\partial h}{\partial r}\right|_R &= \frac{Ae^{R/\lambda} - Be^{-R/\lambda}}{R\lambda} - \frac{Ae^{R/\lambda} + Be^{-R/\lambda}}{R^2} = 0 \\ \rightarrow \quad \frac{B}{A} &= e^{2R/\lambda}\left(\frac{R-\lambda}{R+\lambda}\right) \end{aligned} \quad \text{[S13]}$$

and the inner continuity condition gives:

$$\begin{aligned} h(b) &= \frac{A}{b}\left(e^{b/\lambda} + \frac{B}{A}e^{-b/\lambda}\right) = h_c \\ \rightarrow \quad A &= \frac{(R+\lambda)e^{-b/\lambda} b h_c}{(R+\lambda) + (R-\lambda)e^{2(R-b)/\lambda}} \end{aligned} \quad \text{[S14]}$$

Next we find the steady-state h_c by setting $\frac{dh_c}{dt} = 0$. First, solving for the current across the boundary gives:

$$\begin{aligned} \frac{I}{k_d \rho v_c} &= -\frac{4\pi b^2 \lambda^2}{v_c} \left.\frac{\partial h}{\partial r}\right|_b \\ &= -\frac{4\pi b^2 \lambda^2}{v_c} A\left(\frac{e^{b/\lambda} - \frac{B}{A}e^{-b/\lambda}}{b\lambda} - \frac{e^{b/\lambda} + \frac{B}{A}e^{-b/\lambda}}{b^2}\right) \\ &= h_c z / v_c \end{aligned} \quad \text{[S15]}$$

where the last step comes from plugging in the results for A, $\frac{B}{A}$ and we define:

$$\frac{z}{4\pi b\lambda} = \frac{(\lambda - b)(R+\lambda) + (\lambda+b)(R-\lambda)e^{2(R-b)/\lambda}}{(R+\lambda)+(R-\lambda)e^{2(R-b)/\lambda}} \quad \text{[S16]}$$

Now solving for h_c directly:

$$\begin{aligned} k_u(1-h_c) - I/\rho v_c - k_d h_c &= 0 \\ h_c(1 + k_u/k_d + z/v_c) &= k_u/k_d \\ h_c &= \frac{k_u/k_d}{1 + k_u/k_d + z/v_c} \end{aligned} \quad \text{[S17]}$$

The total material outside of the fixed cluster is then given by:

$$\begin{aligned} S =& (N_0 - \langle n\rangle)\frac{4\pi\left(\int_a^b r^2 h_c dr + \int_b^R r^2 h(r) dr\right)}{4\pi\int_a^R r^2 dr} \\ =& \rho\left(h_c v_c + 4\pi A\left[(r\lambda - \lambda^2)e^{r/\lambda} - \frac{B}{A}(r\lambda+\lambda^2)e^{-r/\lambda}\right]_b^R\right) \\ =& \rho h_c(v_c + z) \end{aligned} \quad \text{[S18]}$$

as in Eq. 3. To get the total material including the fixed cluster, we add the the material per unit at the source ($h_0\ell_0 = 1$) multiplied by the size of the fixed cluster, ($\langle n\rangle - 1$) (ignoring the fixed unit itself). The limiting behaviors for short τ_d, τ_{enc}, and τ_u (indicated in Fig. 3) are each derived straightforwardly through expansion of z for small $\lambda \ll R$.

D. Simulation Methods.

D.1. Dynamic network simulations. We start with the dynamic network simulation framework described in Ref. (5). Edge-units are spherocylinders with steric exclusion radius $r_s = 0.15$ and length $\ell_0 = 0.5$. Using length units of μm, these values correspond to the thickness and fragmented unit volume observed for mitochondria *in vivo* (6–9). The effect of ℓ_0 on network structure and material spreading through the networks is addressed in Section 2E, Fig. S4.

All $N_0 = 250$ units are confined by a reflecting domain boundary of radius $R = 5$. To generate the network structures, we use variable tip-tip and tip-side fusion rate constants k_{u1}, k_{u2} as indicated in the text, with the fission rate constant set to $k_f = 1$ to normalize time units throughout. Fusion between two nodes can occur when the nodes are separated by less than $2r_c$, where $r_c = 0.2$ is the contact radius. Random motion of units and clusters is realized by Brownian forces sampled from a normal distribution with variance $2/\Delta t$ in each dimension, applied at each node, giving $D_1 \approx 0.5$ per isolated unit. Assuming a fission rate on the order of $k_f = 1/(2\text{min})$, this dimensionless diffusivity corresponds to approximately $D_1 \approx 0.25\mu\text{m}^2/\text{min}$, consistent with observations of stochastic mitochondrial motion in mammalian cells (10–12).

Diffusion of material through the network is accomplished using finite-volume simulations built on top of the dynamic network simulation. Material concentration fields are discretized on a per-unit basis, with the change in concentration across each junction (node) in the network calculated as:

$$\frac{dc_{n^i_j}}{dt} = \frac{D_p}{\ell_0^2}\sum_{k=1}^{\deg(i)}(c_{n^i_k} - c_{n^i_j}) \quad \text{[S19]}$$

where $D_p = 4800$ is the default particle diffusivity and $c_{n^i_j}$ is the per-unit concentration on the j-th edge connected to node i. Material exchange is thus only possible across connected (fused) units. Decay is applied uniformly across the network as $dc/dt = -k_d c$. The simulations proceed through forward Euler stepping with a timestep of $\Delta t = 10^{-4}$. After allowing the network structure to equilibrate for 2×10^6 simulation steps, we initialize the concentration of all units at $c = 0$, except for one source unit chosen at random, whose per-unit concentration is fixed to $c = \ell_0 h_0 = 1$. We then run each spreading simulation for at least 5 times the decay time ($5\tau_d$), averaging the total material over the second half of the simulation. We further average over 9 choices of the source node per simulation and at least 3 independent simulation replicates.

D.2. Interacting spheres simulations for social networks. For simulations of the simplified model for highly fragmented mitochondria, we take an agent-based approach with $N_0 = 251$ identical spherical units of steric exclusion radius $a/2 = 0.15$. These spherical units are initially distributed uniformly inside a reflecting spherical boundary of radius $R = 5$ and are allowed to diffuse with diffusivity $D_1 = 0.5$. One unit is selected as the source and is assigned constant concentration $c = 1$. Transient fusion between two units occurs at rate k_u whenever the centers of the spheres are separated by less than b, where $b = 0.4$ represents the contact distance. Upon fusion, the sphere concentrations c_1, c_2 are updated to $c_1' = c_2' = (c_1 + c_2)/2$ to represent equilibration. Fusions are immediately followed by fission. If a non-source unit fuses with the source unit, its concentration is raised to $c = 1$. Decay is applied uniformly across the non-source units with $c(t+\Delta t) = c(t)e^{-\Delta t/\tau_d}$ where the timestep $\Delta t = 10^{-2}\cdot\min(10^{-1}, k_u^{-1}, \tau_d)$. We run each simulation until the simulation time is at least $t = 50$ for $\tau_d \le 1$ and at least $t = 2000$ for $\tau_d > 1$, and average the total material over the last quarter of the simulation.

E. Experimental Imaging and Data Processing.

E.1. Mammalian cell growth, transfection, and vital dyes. Human IMR90 cells (American Type Culture Collection #CCL-186) and U2OS-Cas9 cells (HD Cas9-012) were grown in high-glucose Dulbecco's modified Eagle's medium (DMEM) supplemented with 10% heat-inactivated fetal bovine serum, 1% L-Glutamine (100X), and 1% penicillin/streptomycin at 37°C in a humidified 5% CO2 chamber. Human SH-SY5Y cells (American Type Culture Collection #CRL-2266) were grown in high glucose Dulbecco's modified Eagle's medium (DMEM) supplemented with 10% heat-inactivated fetal bovine serum, 1% non essential amino acids (NEAA) and 1% Sodium Pyruvate (NaPyr) at 37°C in a humidified 5% CO_2 chamber. Before imaging, cells were seeded onto glass-bottom 35-mm dishes (Mattek) and cultured for 24 to 48 hours. Transient transfection was performed using Lipofectamine 2000 according to the manufacturer's protocol (Thermo Fisher Scientific) and cells were imaged 24 hours later, unless otherwise noted. U2OS-Cas9 cells were transfected with non-targeting Scrambled sgRNA#1 (EditCo) using RNAiMAX Transfection Reagent according to the manufacturer's protocol (Thermo Fisher Scientific), media was replaced 24 hours later, and cells imaged 5 days later. For live cell imaging, 1 ml of conditioned cell media was removed and reserved prior to staining with 50 nM MitoTracker Deep Red (Thermo Fisher Scientific) directly in the imaging dish for 30 min. Medium was then replaced with the reserved conditioned media just before imaging. Plasmids and resource identifiers are reported in Table S1.

Plasmid	Source and Reference
Mito-BFP	mito-BFP was a gift from Gia Voeltz (Addgene plasmid # 49151; http://n2t.net/addgene:49151; RRID:Addgene_49151). https://pubmed.ncbi.nlm.nih.gov/21885730/
Su9-EGFP	Su9-EGFP was a gift from David Chan (Addgene plasmid # 23214; http://n2t.net/addgene:23214; RRID:Addgene_23214). https://pubmed.ncbi.nlm.nih.gov/12527753/

Table S1. Plasmids used to generate experimental imaging data.

E.2. Microscopy and image acquisition. All images were acquired using a Zeiss LSM 980 with Airyscan 2 laser scanning confocal microscope, equipped with 405-nm, 488-nm, 561-nm, and 639-nm laser lines and Fast Airyscan detector array. Images were acquired using an inverted 63x/1.4 numerical aperture oil objective. All live imaging was done in a humidified chamber at 37°C and in the presence of 5% CO_2. Airyscan processing was performed using Zeiss ZEN Blue software version 3.7 (Carl Zeiss). Image brightness and/or contrast were linearly adjusted in ZEN Blue. Imaging data has been made publicly available at https://doi.org/10.5061/dryad.cjsxksnkb.

E.3. Network Extraction. Individual cells are separated and manually cropped in ImageJ using a max projection in Z and time. Areas and volumes are estimated by measuring the cell area and subtracting the nuclear area, with height assumed uniform throughout. The mitochondria for each cell are segmented and tracked using the software package Nellie (13). Skeletons and corresponding network structures are extracted from the Nellie segmentation at each time point using the skimage (14) and skan (15) python packages. Degree distributions for the network are computed as in (5) to provide for an assumed underlying mitochondrial unit length of 0.5μm, matching simulations. We then extract all structural parameters (cluster sizes, radius of gyration, fractal dimension, mean graph distance, etc.) for each imaged network in the same way as for simulated networks, using a custom network analysis package in Matlab (16).

E.4. Dynamic measurements. There are two dynamic parameters which must be extracted from experimental data in order to apply the analytic model. From Nellie outputs of segmented and time-linked objects, we extract the trajectories of individual labeled mitochondria, as marked by their center-of-mass positions in consecutive frames. Trajectories containing fewer than three time points or very fast motion of greater than 0.64μm/s are discarded. We restrict our analysis to mitochondria of length 2μm or less, as large clusters are more prone to tracking artifacts. Positions are projected into the xy-plane and mean squared displacements (MSD) are calculated between consecutive frames on each trajectory. Finally, the effective mitochondrial diffusivity for a given cell is estimated as: $D_{\text{mito}} = \langle \Delta r^2 \rangle / (4\Delta t)$, where $\langle \Delta r^2 \rangle$ is the MSD between consecutive frames and Δt is the time between frames.

Second, fission events are manually identified in ImageJ using the max projection in Z for each cell. To calculate the effective per-node fission rate constant, k_f, we then divide these fission counts by the elapsed time for the movie and a weighted sum of junction nodes $(x_2 + \frac{3}{2}x_3)$, with x_i the average number of degree-i nodes in the network (6).

It is important to make a distinction between microscopic fusion and fission events, which occur with local rate constants k_f, k_u, and large-scale events that encompass network rearrangement. The latter include 'novel fusions' (fusion with a new network unit) and 'complete fission' events that proceed them. As found in (5), for simulated mitochondrial networks, the complete fission rate constant is roughly ten-fold lower than the local fission rate constant k_f. Because microscopic fissions are quickly followed by re-fusion and are unlikely to be observed in our time-lapse images, we assume these counts correspond to complete fission events only, and set k_f to be ten-fold higher than the observed rate. Finally, the cluster-cluster fusion rate k_u is estimated as $k_u \approx \langle k_f \rangle (C_1/\delta v_1 \cdot x_1 \left(\frac{x_1}{x_1+x_2}\right) + C_2/\delta v_2 \cdot \frac{2x_1x_2}{x_1+x_2})$ where $C_1 = V_{\text{cell}} x_2 / (\frac{1}{2}x_1^2)$, $C_2 = \frac{3}{2} V_{\text{cell}} x_3 / (x_1 x_2)$, and $\delta v_1, \delta v_2$ are the contact volumes per node for tip-tip and tip-side fusion (5, 6).

We note that our estimates for the local fission rate are likely an underestimate, as we are limited by framerate and the ability to manually identify all fission events within a given movie. Estimates for the mitochondrial diffusivity, on the other

hand, are likely a slight overestimate, as tracking artifacts may cause artificial jumps in trajectories which cannot be completely filtered out. However, these limitations do not alter the results plotted in Fig. 5b,d because the mitochondria in these cells are already in a mobility-limited regime. This means that when spreading occurs via cluster-cluster interactions as described by the social network model, the interaction time is dominated by the timescale to encounter new clusters, τ_{enc}.

We can see quantitatively that the mitochondria in our data set fall into this regime by analyzing the ratio: $\tau_{\text{enc}}/\tau_u = \rho v_c k_u/(\rho 4\pi D b)$, which compares the total fusion rate in the contact zone to the rate of mitochondria arriving at the contact zone. For the cells in our data set, this ratio ranges from $10^0 - 10^3$, with the majority of cells centered around 10^1. Because our estimate for k_u is a lower bound and our estimate for D is an upper bound, the predicted ratio of τ_{enc}/τ_u must also be a lower bound. Therefore we can safely say that the results for spreading in the mitochondrial networks used for this analysis are insensitive to potential inaccuracy in the measured fission rate.

E.5. Solving the social network model in a slab-like geometry. As the cells we are analyzing are nearly flat (area $\sim 1000\mu\text{m}^2$, height $\sim 2\mu\text{m}$), we solved the social model (Eq. 2) in a quasi-2D slablike geometry to make the predictions shown in Fig. 5. This amounts to solving the diffusion equation in cylindrical coordinates, with a result that looks exactly the same as Eq. 3 up to a redefinition of the spatial spreading term, contact volume, and mitochondrial unit density:

$$\frac{z}{2\pi b H \lambda} = \frac{-I_1(b/\lambda)K_1(R/\lambda) + K_1(b/\lambda)I_1(R/\lambda)}{I_0(b/\lambda)K_1(R/\lambda) + K_0(b/\lambda)I_1(R/\lambda)}, \quad \text{[S20a]}$$

$$v_c = \pi(b^2 - a^2)H, \quad \text{[S20b]}$$

$$\rho = (N_0 - \langle n \rangle)/[\pi(R^2 - a^2)H], \quad \text{[S20c]}$$

where I_j, K_j are modified Bessel functions, H is the height of the cell and we calculate $R = \sqrt{A_{\text{cell}}/\pi}$, the effective planar radius of the cell based on the measured cell area. The steric radius a is set based on the radius of gyration averaged over all clusters and b is calculated from Eq. S20b, analogously to the 3D simulations.

In 4 of the analyzed SH-SY5Y cells, the average mitochondrial cluster size was greater than half of the total mitochondrial content in the cell. As the social network model assumes multiple interacting clusters of similar size, we used only the stationary network approximation to estimate the spreading times for these networks, with the source assumed to be located on a cluster of the typical largest size (for these networks, ~ 0.75 of the total mitochondrial content).

**Parameter (unit)**	**SH-SY5Y** (17 cells)	**IMR90** (21 cells)	**U2OS** (12 cells)
cell area, A (μm^2)	849	1190	1580
cell height, H (μm)	2.04	2.04	1.90
cell effective radius, R (μm)	16.4	19.5	22.4
total mitochondrial length (μm)	663	856	1190
mean cluster size (μm)	251	165	231
cluster radius of gyration (μm)	5.11	4.55	4.80
relative mito diffusivity (μm^2/min)	0.592	0.595	0.908
number of tips per cluster, x_1	69.3	43.4	67.0
fractal dimension, d	1.24	1.13	1.18
mean graph distance, $\langle r_g \rangle$ (μm)	20.6	19.5	22.0
mito unit length, ℓ_0 (μm)	0.5	0.5	0.5
mito contact radius, r_c (μm)	0.2	0.2	0.2
mito steric radius, r_s (μm)	0.15	0.15	0.15
tip-tip association constant, ρc_1	19.3	23.8	21.8
tip-side association constant, ρc_2	2.45	2.01	2.18
cluster-cluster steric distance, a (μm)	10.2	9.10	9.60
cluster-cluster contact volume, v_c (μm^3)	29.1	20.5	27.4
cluster-cluster contact distance, b (μm)	10.4	9.27	9.83
number of degree-2 nodes per cluster, x_2	271	199	253
per-node fission rate, k_f (min^{-1})	0.0217	0.0358	0.0151
local cluster-cluster fusion rate, k_u (min^{-1})	165	175	108
d-dimensional cluster size, R_n (μm)	27.7	27.5	30.3
d-dimensional unit size, a_n (μm)	0.182	0.164	0.169

Table S2. Parameters describing mitochondrial structure and dynamics in three human cell types. Top: measured from live-cell imaging data. Each value is averaged across all cells of the given cell type (SH-SY5Y, IMR90, and U2OS cells). Middle: assumed, based on past work (5). Bottom: derived from other parameters, as described in Supplemental Methods.

2. Supplemental Results

A. Run and tumble motion in social networks. Here we test the effect of driven, ballistic runs on spreading rates in the fragmented limit of our model. Taking the same approach as in Fig. 3, we first calculate the steady-state content for a set of

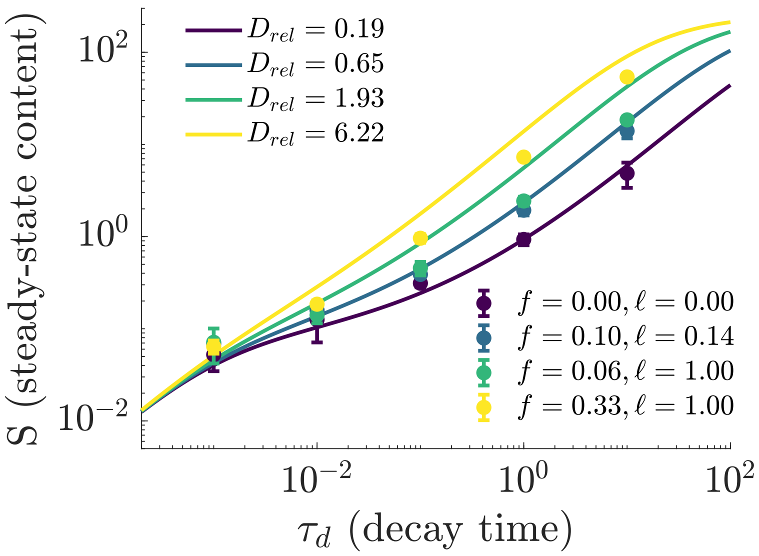

Fig. S1. Increasing unit motility via run-and-tumble motion can enhance network filling. Steady-state network content is plotted as a function of decay times for a fully fragmented set of interacting spherical units, as in Fig. 3. Curves indicate analytic predictions assuming effective diffusive motion (Eq. 3). Dots with error bars indicate simulation results where we incorporated run and tumble dynamics with variable run length (ℓ) and fraction of time spent running (f). Adding short and sparse directed runs (blue) boosts network filling compared to a system with purely passive particles (purple). Short run dynamics are well captured by the analytic model with an appropriate choice of effective diffusivity D_{rel}. When the run length becomes of the same order as the domain size ($\ell = 1$, $R = 5$) the run and tumble units spread material more slowly than would be expected for diffusive units with the same effective diffusivity (green and yellow).

spherical units which are purely diffusive with diffusivity $D_1 = 0.1$. The corresponding relative diffusivity between two units (as used in Eq. 2, 3) is $D = 0.2$. We chose $k_u = 1000$ such that $\tau_u \ll \tau_{\text{enc}}$, placing the system in a mobility-sensitive regime. The resulting network filling curve is plotted in Fig. S1 (purple), with the analytic prediction of Eq. 3 matching the simulations.

Next, we introduce directed run-and-tumble-like motion, parameterized via an on-rate k_{on} to begin a run and an off-rate k_{off} to re-enter a passive diffusive state. While in the on-state, units move with speed $v = 60$ (0.5μm/s in real units) in a direction randomly chosen at the start of the run. Units in the off-state move diffusively with $D_1 = 0.1$. Together, these parameters set the fraction of units in the active state $f = \frac{k_{\text{on}}}{k_{\text{on}}+k_{\text{off}}}$, and their typical run length $\ell = v/k_{\text{off}}$. As noted in prior work (17, 18), the intermittent running and diffusive motion can be described as an effective diffusivity on time-scales much greater than a single run or pause time.

For each simulation, we extract the effective diffusivity of the particles via their mean-squared displacement, as described in Sec. 1C.2, analogous to the analysis of mitochondrial motion in imaging data (Sec. 1E.4). The resulting diffusivity is doubled to give the relative diffusion between two mobile particles (D_{rel}).

The effective diffusivity increases as the running fraction and/or the run length increases. Consequently, the approximate model for fragmented networks predicts more rapid filling as f and ℓ are raised (Fig. S1). So long as the run length remains much smaller than the domain size, the increase in network filling due to higher mobility is well approximated by the effective diffusion model (blue curve in Fig. S1). However, as run lengths become long, the rate of encounter approaches a plateau, and the effective diffusivity overestimates how quickly particles find each other (18). Thus, simulations with $\ell = 1.0$ result in less network filling than predicted by the diffusive encounter model in Eq. 3.

We note that the reported effective diffusivity of small mitochondria (10–12) likely already incorporates short periods of active motion. However, mitochondrial run lengths can vary widely, reaching several micrometers in, *eg*, plant cells (19) and neurons (20). Furthermore, motion along cytoskeletal filaments can introduce correlations in space that may affect the rate at which they encounter each other. The model presented here is valid in a regime where mitochondrial movements can be treated as effectively diffusive. Exploring the consequence of long runs (comparable in size to the cell itself) is a potentially fruitful avenue for future work.

B. Tracking encounters on temporal networks. Here we present results for encounter and interaction times across our simulations. Our approximate analytic model for the partially fragmented regime assumes identical spherical clusters diffusing in a domain much larger than the cluster size. As our clusters do not form perfect, identical spheres, and may approach a substantial fraction of the domain size in the highly fused limit, we sought to verify that this assumption remains accurate across the parameters used.

To compare the encounter times between clusters against the analytic approximation, we ran simulations with identical parameters to those in Fig. 4. After running the structures to steady state, we turned off fusion and fission, recording the first time that each pair of distinct clusters came within contact distance, where fusion would be possible. We then extracted the mean first-encounter time by fitting an exponential to the cumulative distribution function, with individual

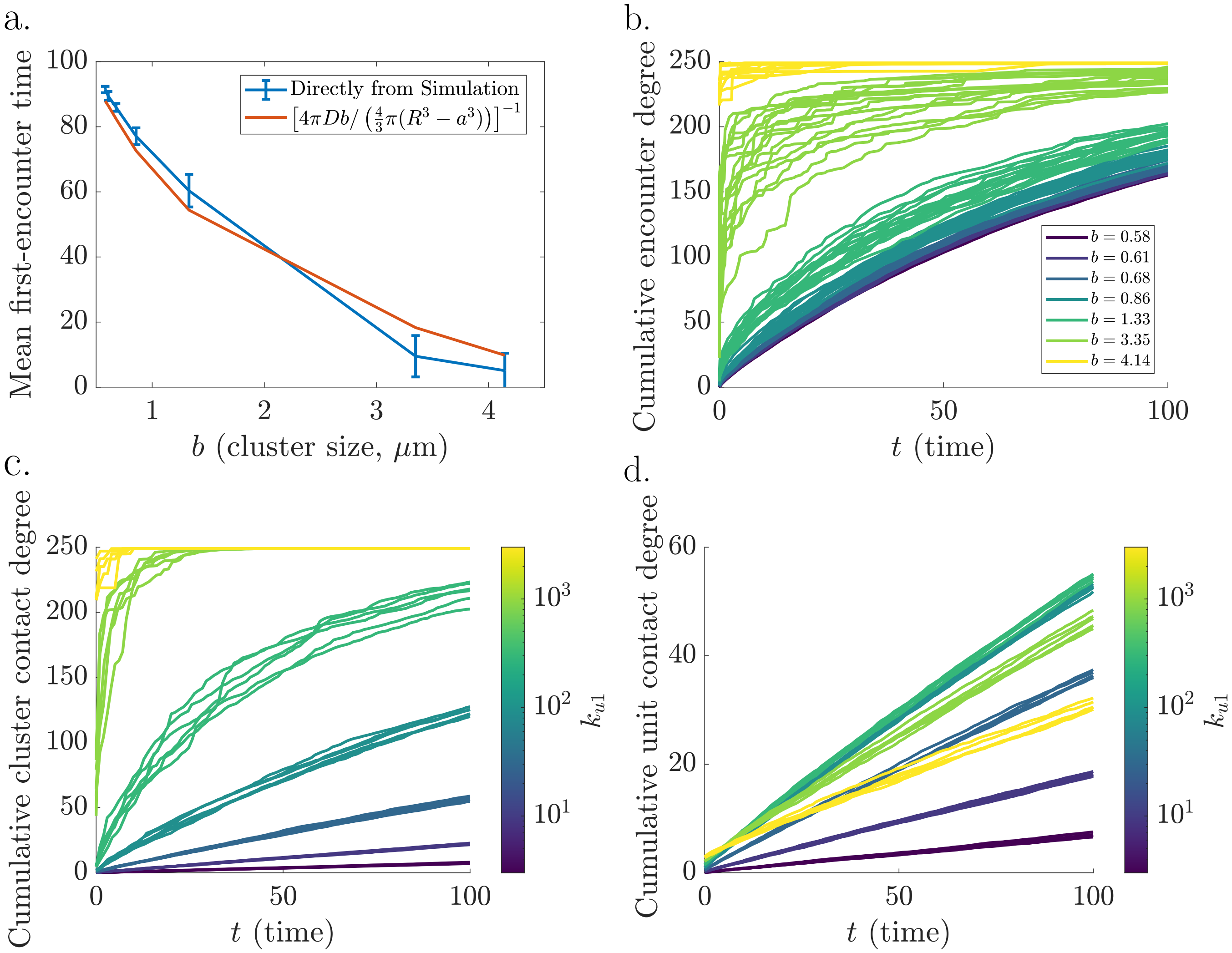

Fig. S2. Tracking encounters and interactions in simulated dynamic networks. (a) The mean first-encounter time between distinct clusters is plotted as a function of b, the cluster-cluster contact distance and compared to the predicted value: $\tau_{\text{enc}} = V/(4\pi Db)$ (21). Here b increases monotonically with the fusion rate k_{u1} while D and $V = 4/3\pi(R^3 - a^3)$ decrease (see Sec 1C.2 for definitions of D, b). (b) The average cumulative unit degree is plotted against simulation time for the same set of simulations. Links are created between all units which are part of the same cluster at $t = 0$, and new links are added between all units in clusters which approach within the contact distance. Repeat encounters between the same clusters do not add additional links. (a,b) use simulations which are initially parameterized identically to those in Fig. 4a with the structure run to steady-state and fusion/fission dynamics subsequently turned off so that clusters maintain their identity over the course of the measurement. (c) For simulations with fusion and fission turned on (identical to Fig. 4a), the cumulative degree is defined by adding links between all pairs of units belonging to clusters that fuse together, accumulated against simulation time. (d) For simulations with fusion and fission on (identical to Fig. 4a), we count the average cumulative number of distinct units that each unit has been directly fused to (shared a node with) over time. On longer timescales, partially connected networks reach higher cumulative degree than either highly fused (yellow) or fragmented (dark blue) networks.

first-encounter times weighted by cluster size. Finally, we compare to the classic result for encounter with an absorbing sphere (21): $1/\tau_{\text{enc}} = 4\pi Db/[4/3\pi(R^3 - a^3)]$ where D, b, R, a are as defined in Supplemental Methods 1C.2. Figure S2a demonstrates good agreement between the predicted and the measured first-encounter times across the range of cluster sizes b (increasing monotonically with k_{u1}) that were tested.

We also investigated the cumulative connectivity for our networks over time, an approach that has been useful in characterizing plant mitochondrial networks (19, 22) as well as other temporal networks built from interacting agents (23). Specifically, we begin with an adjacency matrix at some initial timepoint (after network structure has been equilibrated), describing which units are initially connected. We then step forward in time adding links between units that later become connected while tracking the mean cumulative degree of units over time.

For systems of interacting network clusters, there are several different ways to define temporal connectivity. First, we can add links between all units which are part of the same cluster and add new links between all units in clusters that approach within the contact distance. This definition is based only on spatial proximity and is the closest analogue to prior analyses of plant mitochondrial contacts from imaging data (19, 22). It is relevant for material spreading in a regime where the particle diffusivity within mitochondrial clusters and the local rate of fusion are both infinitely high, so clusters share all their material as soon as they encounter each other. Fig. S2b shows that the resulting cumulative encounter degree is strongly dependent on

the cluster size b. In the highly fused regime, there are very few clusters and they encounter each other rapidly, so that that the encounter degree saturates nearly instantaneously. For the more fragmented regime, the degree increases with time as the clusters explore through space to encounter more distinct encounter partners.

An alternative definition of connectivity requires a successful fusion event to create links between the units of interacting clusters. Fig. S2c demonstrates that the resulting cumulative cluster contact degree is then controlled by the local fusion rate k_{u1}. Again, the cumulative contact degree saturates almost instantaneously when most of the mitochondria are part of a single large cluster.

A third definition of connectivity limits the acumulated links between units to the direct neighbors of each given node. Fig. S2d shows a non-monotonic scaling of the resulting cumulative unit contact degree with increasing fusion rate k_{u1}. At the lowest fusion rates, all links between units are rare, while at the highest fusion rates, most fusions do not result in a novel pairing since the network is more static (5). It is at intermediate fusion rates, where the network is partially fused but continues to rearrange, that the cumulative connectivity is maximized. Notably, this contact degree is particularly relevant for the spreading of particles that move very slowly within a mitochondrial cluster (such as, *eg* nucleoids or inner membrane protein complexes). For such particles, the ability of the mitochondria themselves to move around and find novel interaction partners is important for increasing the overall degree of contacts formed. The non-monotonic dependence of novel fusions on the local fusion rate constant was also noted in our previous work (5). This behavior highlights the interplay between mitochondrial mobility (allowing for large contact degrees) and network connectivity (limited node degree) for enabling the dispersion of different types of particles through the network (25).

C. Time-dependent spread of material without decay. The results in the main text focus on the total material delivered to a network at steady-state, where such a state is reached through a balance of spreading and decay. Simulations and analytical approximations provide a comprehensive picture for this system (Fig. 4). The decay rate k_d in this model plays the role of setting the timescale on which spreading is being considered.

To show the generality of the analytic approximations, we consider the related problem of time-dependent spreading without decay. With $k_d = 0$, there is no steady-state but we can instead quantify the temporal evolution of material content in the network: $S(t)$. To probe this question, we run simulations using the same parameters as in Fig. 4 with $k_d = 0$, tracking the total material delivered to the network over time. Fig. S3 shows that the combined steady-state analytical solution (Eq. 4), provides a good approximation to the time-dependent spreading $S(t)$ when evaluated using the corresponding effective decay rate: $k_d = 1/t$.

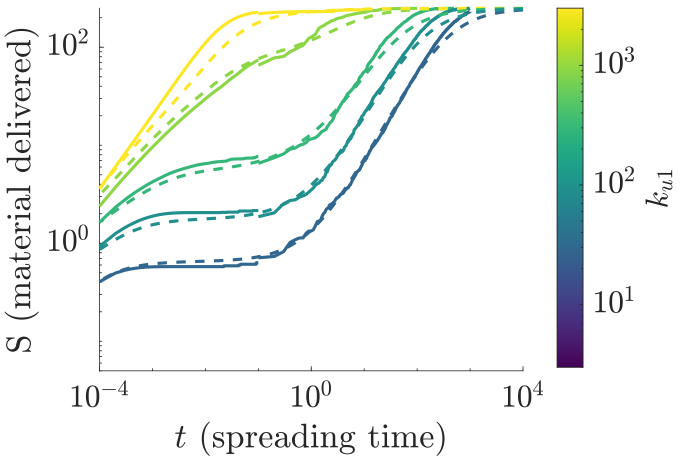

Fig. S3. Total material in the network plotted as a function of time for a system with no material decay, with $S(t = 0) = 0$. Solid lines correspond to simulated networks, filling with material over time. Simulation parameters are identical to Fig. 4 but with $k_d = 0$. Dashed curves give the combined analytic steady-state solution for physical and social networks, as in Fig. 4, plotted with $t = \tau_d$ as the effective spreading time.

D. Estimating dynamic parameters in plant cells. As noted in the main text, mitochondrial networks occupy a wide variety of strucural and dynamic states depending on organism, cell type, growth conditions, etc. For the mammalian cells examined in "Spreading rates on mammalian mitochondrial networks" (main text), we predict spreading through the network to be limited by either material diffusivity (SH-SY5Y cells) or organelle mobility (IMR90 and U2OS cells). A classic study by Arimura et.al. (2004) illustrates an example of a 3rd regime identified by our model in which organelles are highly motile and spatial encounters are frequent, but fusions happen in only a small fraction of encounter events. In their experiment, the authors use a photoconvertible mitochondrial marker to track the dispersion of mitochondrial contents in onion bulb epidermal cells. Specifically, they initially photoconvert mitochondria in one half of the cell, observing a global mixing time between green and red-tagged mitochondria of about 2 hours (26). Importantly, intermediate snapshots (their Figure 3a) show both green and

red mitochondria scattered throughout the domain, indicating that the timescale for fusion is substantially slower than the timescale for spatial exploration ($\tau_{\text{enc}} \ll \tau_{\text{u}}$).

Using the 30-minute image from Arimura et al, we estimate a mean squared displacement on the order of $10^2\mu\text{m}^2$ for individual mitochondrial fragments. This translates to $D_1 = 10^2/(4 \cdot 1800\text{sec}) \approx 0.014\mu\text{m}^2/s$ where the mitochondria are assumed confined to move in 2 dimensions. We further estimate a mitochondrial contact distance of $b \sim 0.6\mu$m and a number density of $\rho \sim 30/300\mu\text{m}^2$. This gives the 2-dimensional encounter timescale (18):

$$\tau_{\text{enc}} \approx \frac{1}{2\pi(2D_1)\rho}\left[\ln\left(\frac{1}{b\sqrt{\pi\rho}}\right) - 3/4\right] \approx 19s, \qquad \text{[S21]}$$

which is far faster than the content mixing (interaction) timescale of about 2 hours observed in the experiment. This encounter timescale also agrees with measurements by Chustecki et al. (19), showing that the mean degree of *Arabidopsis* mitochondria in tracked encounter networks increases at a rate of $\sim 1/20$s before plateauing at longer times. Since these mitochondria are exclusively in small fragments, we can ignore the cluster-filling timescale and approximate $\tau_u \approx \tau_{\text{int}} = 120$min. This allows us to estimate the local fusion rate k_u:

$$k_u = \frac{1}{\rho v_c \tau_u} = \frac{1}{\rho\pi(b^2 - a^2)\tau_u} \approx 0.004\text{s}^{-1} \qquad \text{[S22]}$$

where we estimate $a = 0.5\mu$m. If there is in fact a larger difference between the contact distance b and steric exclusion radius a, the contact volume would increase and the estimated fusion rate would decrease further still. This rate is dramatically smaller than the local fusion rate we extracted for mammalian cells of $2 - 3\text{s}^{-1}$, implying that plant mitochondria may be less likely to undergo fusion and content mixing when coming into spatial proximity.

E. Effect of varying the unit length. The interplay of the kinetic fusion and fission rates as well as the length ℓ_0 of spherocylindrical units combine to determine structural properties of the network. While similar structures can be achieved with different combinations of parameters, here we consider the effect of varying the unit length only, without adjusting fusion and fission rate constants to compensate. Unsurprisingly, increasing the unit length increases the typical separation between junctions, so that the length of linear snake-like segments for a given cluster size is higher, corresponding to a lower graph dimension for the network (Fig. S4a). This work is focused on spreading of material across networks rather than the structure of the networks themselves, so that the altered unit length is simply treated as a shift in the structural space of possible networks.

We extract structural properties for networks with different ℓ_0 values as described in the main text and apply our general model for network filling from a source (Eq. 4). Fig. S4b-c shows that the spreading behavior is qualitatively unchanged when the unit length is varied.

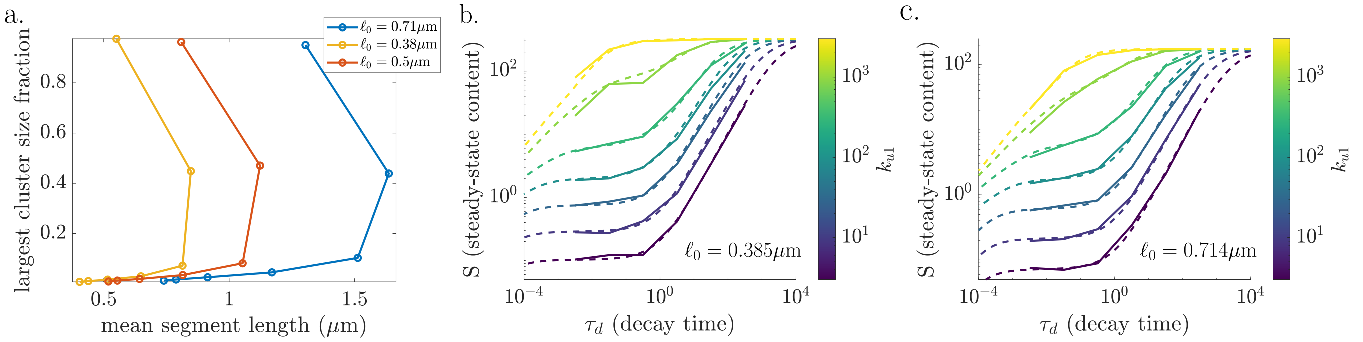

Fig. S4. The analytic model correctly predicts network filling rates when the simulated unit length is varied. (a) The linear segment length and the largest cluster size are plotted together for simulations with increasing fusion rate constant k_{u1}. The ratio k_{u2}/k_{u1} and all other parameters are held constant. Colored curves correspond to three different values of ℓ_0. The total system length is held constant by adjusting the number of units ($N = L/\ell_0$ for fixed L). (b,c) The steady-state material content is plotted as a function of the decay time for different fusion rate constants k_{u1} and two different unit lengths. We include results from simulations (solid lines) and analytics (dashed curves), as in Fig. 4. (b) $\ell_0 = 0.385\mu$m. (c) $\ell_0 = 0.714\mu$m.

3. Supplemental Video Captions

Movie S1. Steady-state dynamics of the mitochondrial network model. Material concentration is indicated on a white-to-red gradient. A single fixed-concentration unit serves as the source. Top: disconnected network, Middle: network near percolation transition, Bottom: hyperfused network. Simulation parameters correspond to those listed in Fig. 1b. Each movie runs over a simulated time of 60 dimensionless units (corresponding to $\approx$ 120min).

Movie S2. Example images of mitochondrial networks in SH-SY5Y (top), IMR90 (middle), and U2OS (bottom) cells. Raw (white) and segmented (multicolored) mitochondria are shown, with all segmentations

obtained using Nellie (13). Connected clusters are indicated by different colors in the initial frame, with subsequent voxel colors determined via Nellie's flow mapping algorithm. All movies run backward (original image) then forward (segmented image) over a span of 116 seconds.

References

1. SR Dunbar, The average distance between points in geometric figures. *Coll Math J* **28**, 187–197 (1997).
2. O Bénichou, C Chevalier, J Klafter, B Meyer, R Voituriez, Geometry-controlled kinetics. *Nat Chem* **2**, 472–477 (2010).
3. S Redner, *A Guide to First-Passage Processes.* (Cambridge University Press), (2001).
4. DJ Smith, MK Vamanamurthy, How small is a unit ball? *Math Mag* **62**, 101–107 (1989).
5. KB Holt, J Winter, S Manley, EF Koslover, Spatiotemporal modeling of mitochondrial network architecture. *PRX Life* **2**, 043002 (2024).
6. VM Sukhorukov, D Dikov, AS Reichert, M Meyer-Hermann, Emergence of the mitochondrial reticulum from fission and fusion dynamics. *PloS Comput. Biol* **8**, e1002745 (2012).
7. SM Rafelski, et al., Mitochondrial network size scaling in budding yeast. *Science* **338**, 822–824 (2012).
8. A Kaasik, D Safiulina, A Zharkovsky, V Veksler, Regulation of mitochondrial matrix volume. *Am J Physiol-cell Ph* **292**, C157–C163 (2007).
9. S Jakobs, CA Wurm, Super-resolution microscopy of mitochondria. *Curr Opin Chem Biol* **20**, 9–15 (2014).
10. Z Wang, et al., MitoTNT: Mitochondrial temporal network tracking for 4D live-cell fluorescence microscopy data. *PLoS Comput. Biol* **19**, e1011060 (2023).
11. B Corci, O Hooiveld, AM Dolga, C Åberg, Extending the analogy between intracellular motion in mammalian cells and glassy dynamics. *Soft Matter* **19**, 2529–2538 (2023).
12. DH Jang, SC Seeger, ME Grady, FS Shofer, DM Eckmann, Mitochondrial dynamics and respiration within cells with increased open pore cytoskeletal meshes. *Biol. Open* **6**, 1831–1839 (2017).
13. AE Lefebvre, et al., Nellie: automated organelle segmentation, tracking and hierarchical feature extraction in 2d/3d live-cell microscopy. *Nat Methods* **22**, 751–763 (2025).
14. S Van der Walt, et al., scikit-image: image processing in python. *PeerJ* **2**, e453 (2014).
15. J Nunez-Iglesias, AJ Blanch, O Looker, MW Dixon, L Tilley, A new python library to analyse skeleton images confirms malaria parasite remodelling of the red blood cell membrane skeleton. *PeerJ* **6**, e4312 (2018).
16. EF Koslover, ZC Scott, networktools (https://github.com/lenafabr/networktools) (2024).
17. JP Rodríguez, M Paoluzzi, D Levis, M Starnini, Epidemic processes on self-propelled particles: Continuum and agent-based modeling. *Phys Rev Res* **4**, 043160 (2022).
18. L Teryoshin, M Hidalgo-Soria, EF Koslover, Encounter times of intermittently running particles. *arXiv preprint arXiv:2512.13973* (2025).
19. JM Chustecki, DJ Gibbs, GW Bassel, IG Johnston, Network analysis of arabidopsis mitochondrial dynamics reveals a resolved tradeoff between physical distribution and social connectivity. *Cell Syst* **12**, 419–431 (2021).
20. BRJ Narayanareddy, S Vartiainen, N Hariri, DK O'Dowd, SP Gross, A biophysical analysis of mitochondrial movement: differences between transport in neuronal cell bodies versus processes. *Traffic* **15**, 762–771 (2014).
21. HC Berg, EM Purcell, Physics of chemoreception. *Biophys J* **20**, 193–219 (1977).
22. K Giannakis, JM Chustecki, IG Johnston, Exchange on dynamic encounter networks allows plant mitochondria to collect complete sets of mitochondrial dna products despite their incomplete genomes. *Quant. Plant Biol.* **3**, e18 (2022).
23. MC González, PG Lind, HJ Herrmann, System of mobile agents to model social networks. *Phys Rev Lett* **96**, 088702 (2006).
24. P Holme, J Saramäki, Temporal networks. *Phys Rep* **519**, 97–125 (2012).
25. JM Chustecki, IG Johnston, Collective mitochondrial dynamics resolve conflicting cellular tensions: From plants to general principles in *Seminars in cell & developmental biology.* (Elsevier), Vol. 156, pp. 253–265 (2024).
26. Si Arimura, J Yamamoto, GP Aida, M Nakazono, N Tsutsumi, Frequent fusion and fission of plant mitochondria with unequal nucleoid distribution. *P Natl Acad Sci* **101**, 7805–7808 (2004).
